# Fermentation in the Human Mouth

**Published:** 1884-02

**Authors:** 


### FERMENTATION IN THE HUMAN MOUTH.

We need not urge every physiologist among the readers of the INDEPENDENT PRACTITIONER, to carefully read Dr. Miller's papers on Fermentation in the Human Mouth. There are some new and interesting facts given this month, that for the first time harmonize known conditions with demonstrated physiological law. The existence of sugar at nearly all times in the oral cavity naturally follows from the presence of starch and the diastatic action of ptyaline, and its conversion into an acid by means of a proper ferment is a natural consequence. That ferment Dr. Miller seems to have demonstrated. Softened dentine will, as he says, take up the sugar with the ferment as would a sponge, and within the mass the acid conversion will go on, with the possible effect of extending the decalcification of tooth tissue. It remains for the succeeding papers to determine the action of this acid upon dentine and the analysis, of it and the chapter on the physiology of the mouth will be looked for with interest.

The supporters of the acid theory of caries have always claimed that the testing of the oral secretions is no sure indication of the reaction on the teeth, because the acids were most energetic while "nascent." Dr. Miller gives a more reasonable explanation of the puerility of the usual saliva tests. It would be gratifying to know whether any such fungus may be found in the mouths of such animals as are not subject to tooth caries, but whose food is, to a greater or less extent, amylaceous.

The record of experiments in this number shows them to have been conducted with faithful care as to details, and in a purely scientific manner, and they are related with a directness and simplicity that reminds one strongly of Tyndall. We are of the opinion that, from the time of the publication of this series of papers, the study of oral physiology will proceed in a different direction, and that these discoveries will mark a new era in dental etiology. For the first time we have a definitely determined point of departure, and an established law of action under which we may with some hope of certainty trace the progress of dental diseases.

